# Differential effects of socioeconomic status on working and procedural memory systems

**DOI:** 10.3389/fnhum.2015.00554

**Published:** 2015-10-08

**Authors:** Julia A. Leonard, Allyson P. Mackey, Amy S. Finn, John D. E. Gabrieli

**Affiliations:** Department of Brain and Cognitive Sciences and McGovern Institute for Brain Research, Massachusetts Institute of TechnologyCambridge, MA, USA

**Keywords:** working memory, procedural memory, socioeconomic status, caudate, hippocampus

## Abstract

While prior research has shown a strong relationship between socioeconomic status (SES) and working memory performance, the relation between SES and procedural (implicit) memory remains unknown. Convergent research in both animals and humans has revealed a fundamental dissociation, both behaviorally and neurally, between a working memory system that depends on medial temporal-lobe structures and the dorsal lateral prefrontal cortex (DLPFC) vs. a procedural memory system that depends on the basal ganglia. Here, we measured performance in adolescents from lower- and higher-SES backgrounds on tests of working memory capacity (complex working memory span) and procedural memory (probabilistic classification) and their hippocampal, DLPFC, and caudate volumes. Lower-SES adolescents had worse working memory performance and smaller hippocampal and DLPFC volumes than their higher-SES peers, but there was no significant difference between the lower- and higher-SES groups on the procedural memory task or in caudate volumes. These findings suggest that SES may have a selective influence on hippocampal-prefrontal-dependent working memory and little influence on striatal-dependent procedural memory.

## Introduction

There has been growing interest in the fields of neuroscience and psychology to understand how socioeconomic status (SES) influences neural and cognitive development in children and adolescents (Hackman and Farah, 2009; Mackey et al., 2015; Noble et al., 2015). SES is a measure of one's overall status in society and can be operationalized by parental income, occupation, education, or a composite of these measures. Most studies examining SES associations with cognitive development have focused on explicit memory tests and found that children from lower-SES backgrounds perform worse on measures of working and declarative memory than their higher-SES peers (Herrmann and Guadagno, 1997; Farah et al., 2006; Noble et al., 2007; Evans and Schamberg, 2009; Sarsour et al., 2011; Hackman et al., 2015). However, it is unknown as to whether SES is associated with differences in procedural (implicit) memory. Convergent research with humans and animals has revealed a fundamental dissociation, both in behavior and in the underlying neural circuitry, between explicit or declarative memory and implicit procedural memory (Squire, 1992; Gabrieli, 1998), raising the possibility that SES may not affect these two memory systems equally. Here we asked whether SES also influences behavioral and neural correlates of procedural memory.

Declarative memory and procedural memory rely on separable neural substrates (Cohen and Squire, 1980; Graf and Schacter, 1985; Knowlton et al., 1996). Long-term declarative memory, measured by performance on explicit tests of recall and recognition, depends upon structures in the medial temporal lobe (MTL) and dorsal lateral prefrontal cortex (DLPFC). Bilateral MTL injury results in global amnesia (Scoville and Milner, 1957; Squire and Zola-Morgan, 1991) and prefrontal lesions impair declarative memory for contextual details (Schacter et al., 1984; Janowsky et al., 1989; Milner et al., 1991). In contrast, simple short-term memory maintenance, operationalized as tests of immediate recall for digits and spatial locations, remains intact after MTL lesions (Scoville and Milner, 1957; Baddeley and Warrington, 1970; Cave and Squire, 1992; Buffalo et al., 1998) and appears to depend upon modality-specific posterior neocortices (Kimura, 1963; Warrington et al., 1971).

Complex working memory capacity, the amount of goal-relevant information that can be simultaneously stored and manipulated, is measured via declarative or explicit tests, and appears to depend upon both MTL and DLPFC brain regions. Complex working memory is impaired in patients with MTL lesions (Hannula et al., 2006; Nichols et al., 2006; Olson et al., 2006a,b; Hartley et al., 2007; Ezzyat and Olson, 2008; Olsen et al., 2012) and also in patients with DLPFC lesions (Barbey et al., 2013). Thus, complex working memory, like declarative memory, appears to depend upon the integrity of a memory system that involves both MTL and DLPFC regions.

Procedural memory, measured implicitly by skill learning over time, does not depend upon the MTL structures supporting declarative and complex working memory, but rather depends upon the integrity of the basal ganglia. Amnesic patients with MTL or similar lesions and severe impairments in declarative memory exhibit intact procedural memory on motoric, perceptual, and cognitive tasks (Milner, 1962; Corkin, 1968; Cohen and Squire, 1980; Gabrieli et al., 1993; Knowlton et al., 1994, 1996). Patients with striatal injuries, however, exhibit impaired learning on such tasks (Heindel et al., 1989; Knowlton et al., 1996; Shohamy et al., 2004).

The neurobiological distinction between MTL-dependent declarative memory and striatum-dependent procedural memory has been evident in the probabilistic classification task, in which participants learn how to classify stimuli into categories (e.g., sunny or rainy weather) based on trial-by-trial feedback. Amnesic patients with MTL lesions and severe declarative memory impairments have exhibited intact learning on this task (Knowlton et al., 1996). In contrast, despite superior declarative memory relative to the amnesic patients, patients with striatal dysfunction due to Parkinson's disease have exhibited severely impaired probabilistic learning (Knowlton et al., 1996; Shohamy et al., 2004). Functional neuroimaging studies with healthy individuals support the conclusion that probabilistic learning is associated with the striatum, specifically the caudate (Poldrack et al., 1999, 2001; Seger and Cincotta, 2005).

Lower-SES has been associated with smaller hippocampal volume (Hanson et al., 2011; Noble et al., 2012, 2015), but the specific mechanisms that mediate this association are uncertain. Lower-SES is a multifaceted construct that includes increased exposure to stress, poor nutrition, and lack of cognitive stimulation (Hackman et al., 2010). All of these factors have been individually linked to the hippocampal integrity in animal experiments. Stress (Sapolsky, 1996) and malnutrition of protein-energy, iron, and zinc harm the developing hippocampus (Georgieff, 2007), while environmental enrichment increases dendritic branching and synaptic density in the hippocampus (Kempermann et al., 1997). Thus, multiple facets of lower-SES may harm development of the hippocampus in humans. On the other hand, there is little evidence about the influence of these factors on the development of striatal structures.

Given that complex working memory and procedural memory rely on separable neural substrates, SES may not affect both systems equally. In the current study, we tested this hypothesis by examining the performance of adolescents from lower- and higher-SES environments (operationalized by family income) on tests of working memory (complex working memory span) and procedural memory (probabilistic classification task). We also measured the volumes of the hippocampus and DLPFC critical for complex working memory, and the caudate, critical for procedural memory.

## Methods

### Participants

As part of a larger study looking at SES, brain development, and educational outcomes (see Mackey et al., 2015; Finn et al., under revision), adolescents were recruited from a variety of home and schooling environments directly through schools, or through summer camps, outreach programs, and advertisements in local papers and on websites. In total, neuroimaging data are presented here for 58 participants (mean age: 14.42, range 13.08–15.18; 27 males; the same 58 from Mackey et al., 2015, and a subset from Finn et al., under revision). Three participants were excluded for the following reasons: one participant had no information on family income, one participant had abnormal brain structure, and one participant had excessive motion artifacts (see “Structural analysis”). This study was approved by the Committee for the Use of Humans as Experimental Subjects at the Massachusetts Institute of Technology. All participants provided written assent, and their parents provided written consent.

Participants were divided into lower-SES and higher-SES groups based on whether or not they had received free or reduced price lunch within 3 years before participation in the study. Participants who were eligible for free or reduced price lunch had a family income below 185% of the poverty line, which, at the time of the study, was approximately $42,000 per year for a family of two adults and two children. Twenty-three adolescents were in the lower-SES group (seven males, 22% African American, 4% Asian, 54% White, 4% Native Hawaiian or Pacific Islander, 26% multiple races, 35% did not report race; 35% not Hispanic, 65% Hispanic) and 35 adolescents were in the higher-SES group (20 males, 6% African American, 14% Asian, 54% White, 3% Native Hawaiian or Pacific Islander, 17% multiple races, 6% did not report race; 91% not Hispanic, 3% Hispanic, 6% did not report ethnicity). The groups did not differ in their distribution of age [lower-SES: *M* = 14.35, *SD* = 0.47; higher-SES: *M* = 14.47, *SD* = 0.38; *t*_(56)_ = 1.05, *p* = 0.30], but did differ in their distribution of boys and girls [*X*^2^(1, *N* = 58) = 3.98, *p* = 0.05], so we controlled for sex in all analyses.

### Procedure

#### Behavioral data acquisition

Participants were tested individually at the Massachusetts Institute of Technology. Participants underwent scanning and behavioral testing during the same visit. The behavioral tests were conducted outside of the scanner. Not all participants were able to complete both memory tasks due to fatigue or reaching the time limit of their visit. Of the 23 lower-SES participants, 19 completed the procedural memory task and 23 completed the working memory task. Of the 35 higher-SES participants, 28 completed the procedural memory task, and 34 completed the working memory task.

#### Income status

With family consent, free/reduced price lunch statistics were obtained from a database maintained by the Massachusetts Department of Elementary and Secondary Education. None of the participants in the current study were enrolled in special education or had limited English proficiency during the 3 years for which data were available.

#### Procedural memory task

A probabilistic classification task (similar to that described in Knowlton et al., 1994) was administered using Psychopy software (Peirce, 2007). Participants were presented with 1–3 of four differently patterned cards on a computer screen and asked to predict whether the cards indicated rain or shine. Card position did not vary across trials. The weather outcome (rain or shine) was calculated according to the conditional probabilities of each card and the combination of cards. For example, a particular combination of three cards was associated with rain 80% of the time. In total, there were 14 card combinations. There were 100 trials, with sunny and rainy outcomes occurring equally overall. Participants had unlimited time to choose an outcome. After the participant chose an outcome, they were given feedback (smiling face for correct responses, sad face for incorrect responses).

A participant was considered to have made an optimal (correct) response if they selected the outcome most often associated with the cue pattern, regardless of the actual probabilistically determined response on the given trial. For example, if a participant selected rain for a card that predicted rain 75% of the time, they would be counted as making an optimal response even if on that particular trial the card predicted sun.

#### Working memory task

A count span task (Conway et al., 2005; Cowan et al., 2005) was administered using Psycopy software (Peirce, 2007). In this task, similar to that described in Finn et al. (2014) participants were presented with consecutive arrays of blue circles, blue triangles, and red circles. They were told to count only the blue circles in each array (ranging from 1 to 9 circles in each array) and to hold that number in mind. They could press the space bar to proceed with the next array, or it would forward to the next display after 5 s. Loads ranged from 1 to 6 and were presented in random order, for a total of three instances of each load. After the presentation of arrays, participants were prompted to enter the number of blue circles in the order they were presented. Participants were given full credit for a given load if they got two out of three instances correct and half credit for each load they got one out of three instances correct (Daneman and Carpenter, 1980).

#### Neuroimaging data acquisition

As described in Mackey et al. (2015), structural MRI data were acquired at the Athinoula A. Martinos Imaging Center at the McGovern Institute for Brain Research at the Massachusetts Institute of Technology. Data were acquired using a 32-Channel Tim Trio 3 Tesla, high-speed magnetic resonance imaging (MRI) scanner equipped for echo planar imaging (EPI; Siemens, Erlangen, Germany). An automated scout image was acquired and shimming procedures were performed to optimize field homogeneity. A multi-echo high-resolution structural image was acquired using a protocol designed for children to better account for motion artifacts (repetition time = 2530 ms; echo times = 1.64, 3.44, 5.24, 7.04 ms; flip angle = 7°; resolution = 1 mm isotropic; Tisdall et al., 2012).

#### Structural analysis

As described in Mackey et al. (2015), data were visually inspected for image quality by two coders blind to income group. Using a visual guide of artifacts associated with motion, coders rated the images on a scale of one (perfect) to four (unusable). If coder ratings differed by more than a point, a third blind coder made a final decision. One participant was excluded for poor image quality. Ratings did not differ between the lower- and higher-SES groups [lower-SES: *M* = 2.04, *SD* = 0.45; higher-SES: *M* = 2.04, *SD* = 0.43; *t*_(56)_ = −0.05, *p* = 0.96].

The hippocampus was segmented by software from Iglesias et al. (2015) that uses Bayesian inference to apply a manually labeled hippocampal atlas to MRI images. The first author, blind to participant income group, manually checked all hippocampal segmentations. At the time of analysis, this software was only available for segmentation of the hippocampus, so the first author, blind to participant income, manually edited each caudate volume from FreeSurfer 5.3′s automated subcortical segmentation. Although FreeSurfer 5.3 does not have highly accurate subcortical parcellations, its cortical parcellations are well-validated (Klein et al., 2010). Therefore, DLPFC volumes were taken from FreeSurfer 5.3 parcellations (Fischl et al., 2002, 2004). Structural analyses controlled for sex because brain anatomy has been shown to differ by sex (e.g., Lenroot et al., 2007).

## Results

### Memory performance

**Figure 1:**
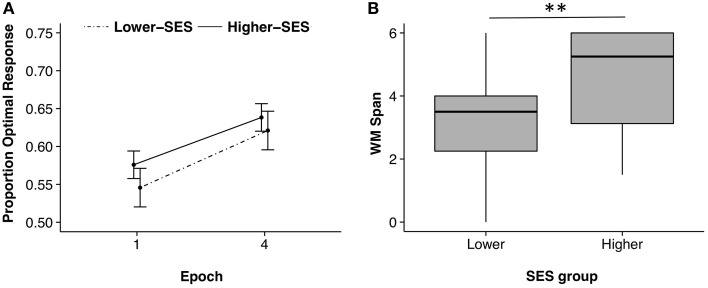
**Probabilistic classification and working memory performances for lower-SES and higher-SES groups**. **(A)** Learning on the probabilistic classification task shown through improved accuracy from the first 25 trials, epoch 1, to the last 25 trials, epoch 4. Error bars corrected for within subjects design. **(B)** Working memory (WM) span by SES group; ^**^p < 0.01.

Both the procedural memory learning score (the difference between the proportion of trials on which the optimal response was chosen on the last 25 trials vs. the first 25 trials) and the count span score were Z-scored so that they could be compared statistically. A repeated measures analysis of variance (ANOVA) revealed a significant SES group by task interaction [*F*_(1, 97)_ = 5.00, *p* = 0.03, η^2^ = 0.05], a main effect of SES [*F*_(1, 97)_ = 5.35, *p* = 0.02, η^2^ = 0.05], but no main effect of task [*F*_(1, 97)_ = 0.003, *p* = 0.96, η^2^ = 0]. Planned *post-hoc* linear model group comparisons showed that the lower-SES group and higher-SES group did not significantly differ on their procedural memory learning score [lower-SES: *M* = 0.08, *SD* = 0.22; higher-SES: *M* = 0.06, *SD* = 0.19; *b* = 0.02, *t*_(44)_ = 0.31, *p* = 0.76, *r*^2^ = −0.04, 95% CI [−0.11, 0.14]; Figure 1A]. Further, a repeated measures ANOVA on optimal performance over the four epochs (25 trials per epoch) showed a significant main effect of epoch, demonstrating learning during the probabilistic classification task [*F*_(3, 135)_ = 3.42, *p* = 0.02, η^2^ = 0.07], but no significant main effects of SES group [*F*_(1, 44)_ = 0.36, *p* = 0.55, η^2^ = 0.008], and no epoch by SES interaction [*F*_(3, 135)_ = 0.18, *p* = 0.91, η^2^ = 0.003].

Data from the count span task were not normally distributed (Shapiro–Wilk test *p* < 0.05), so we performed a Kruskal–Wallis H-test. Planned *post-hoc* analyses showed that working memory capacity was significantly smaller in the lower-SES than the higher-SES group [lower-SES: Median = 3.50; higher-SES: Median = 5.25; *X*^2^(1) = 8.72, *p* = 0.003; Figure 1B].

### Neuroanatomical volumes

**Figure 2:**
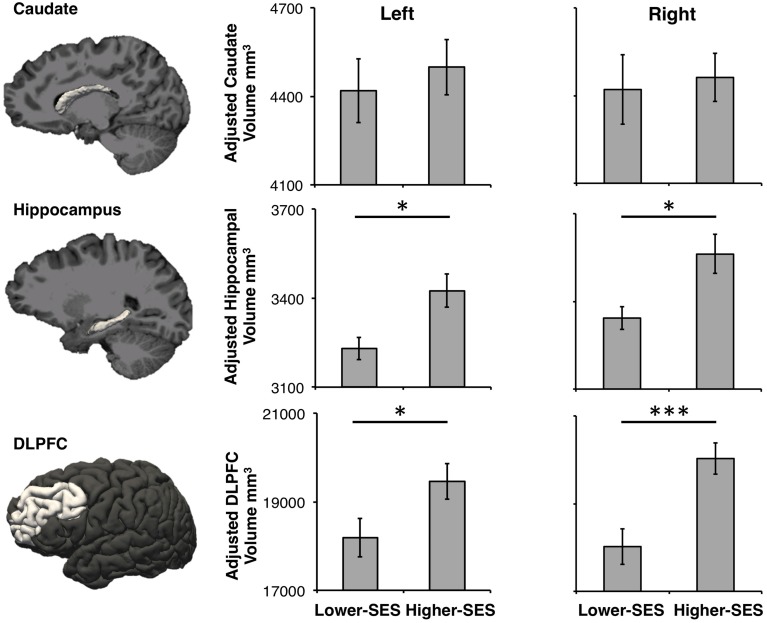
**Caudate, hippocampus, and DLPFC volumes in lower-SES and higher-SES groups**. All volumes are adjusted for sex. Error bars represent standard error; ^*^p < 0.05; ^***^p < 0.001.

Volume data were Z-scored to account for large differences in volume across the hippocampus, caudate, and DLPFC, so that they could be statistically compared. A repeated measures ANOVA on volume showed a main effect of SES [*F*_(1, 335)_ = 50.09, *p* < 0.001, η^2^ = 0.42], region [*F*_(1, 335)_ = 3.77, *p* = 0.02, η^2^ = 0.06], and an SES by region interaction [*F*_(1, 335)_ = 7.14, *p* < 0.001, η^2^ = 0.12]. Planned *post-hoc* t-tests were conducted on each region by SES group. Neither right nor left caudate volumes differed significantly between the SES groups [Right: *b* = 42.47, *t*_(55)_ = 0.30, *p* = 0.77, *r*^2^ = 0.01, 95% CI [−242.61, 327.55]; Left: *b* = 83.92, *t*_(55)_ = 0.57, *p* = 0.57, *r*^2^ = 0.03, 95% CI [−212.82, 380.67]]. Both left and right hippocampus volumes were significantly smaller in the lower-SES group than the higher-SES group [Right: *b* = 242.80, *t*_(55)_ = 2.57, *p* = 0.01, *r*^2^ = 0.19, 95% CI [53.25, 432.35]; Left: *b* = 216.31, *t*_(55)_ = 2.64, *p* = 0.01, *r*^2^ = 0.23, 95% CI [52.16, 380.45]]. Further, both left and right DLPFC volumes were significantly smaller in the lower-SES group than the higher-SES group [Right: *b* = 2359.54, *t*_(55)_ = 3.80, *p* < 0.001, *r*^2^ = 0.48, 95% CI [1116.12, 3602.96]; Left: *b* = 1379.20, *t*_(55)_ = 2.15, *p* = 0.04, *r*^2^ = 0.33, 95% CI [94.81, 2663.55]; Figure 2]. This same pattern held within the subset of participants who completed both the declarative and procedural tasks [Right caudate: *b* = 28.54, *t*_(44)_ = 0.19, *p* = 0.84, *r*^2^ = 0.03, 95% CI [−270.57, 327.65]; Left caudate: *b* = 79.69, *t*_(44)_ = 0.52, *p* = 0.61, *r*^2^ = 0.06, 95% CI [−231.18, 390.55]; Right hippocampus: *b* = 224.87, *t*_(44)_ = 2.13, *p* = 0.04, *r*^2^ = 0.18, 95% CI [12.05, 437.69]; Left hippocampus: *b* = 234.64, *t*_(44)_ = 2.56, *p* = 0.01, *r*^2^ = 0.25, 95% CI [50.19, 419.08]; Right DLPFC: *b* = 2121.65, *t*_(44)_ = 3.63, *p* < 0.001, *r*^2^ = 0.59, 95% CI [943.74, 3299.56]; Left DLPFC: *b* = 1216.30, *t*_(44)_ = 2.24, *p* = 0.03, *r*^2^ = 0.53, 95% CI [112.63, 2310.00]].

Exploratory analyses examined the relations between each ROI volume and behavioral performance on the procedural and working memory task within each SES-group. Within the higher-SES group, greater right hippocampal volume predicted greater working memory [*b* = 0.001, *t*_(31)_ = 2.10, *p* = 0.04, *r*^2^ = 0.08, 95% CI [0.00004, 0.003]], and smaller left caudate volume predicted greater procedural memory learning [*b* = −0.0002, *t*_(25)_ = −2.38, *p* = 0.03, *r*^2^ = 0.14, 95% CI [−0.0003, −0.00002]]. Within the lower-SES group, smaller left DLPFC volume predicted greater procedural memory learning [*b* = −0.00007, *t*_(25)_ = −2.80, *p* = 0.01, *r*^2^ = 0.37, 95% CI [−0.0001, −0.00002]]. No models reached significance after FDR correction for the 12 comparisons made within each SES group.

## Discussion

SES was differentially associated with the behavioral and neural correlates of working and procedural memory. Lower-SES adolescents had worse working memory (reduced complex working memory span), but equivalent procedural memory (probabilistic classification learning) compared to the higher-SES adolescents. This behavioral disparity was reflected in the neural structures supporting these memory systems: hippocampal and DLPFC volumes (critical for working memory) were larger in higher-SES adolescents, whereas caudate volumes (critical for procedural memory) did not differ between the groups. This is the first study to show that SES selectively affects hippocampal-prefrontal-dependent working memory, while not affecting striatal-dependent procedural memory.

The findings that lower-SES was associated with reduced working memory and reduced hippocampal and DLPFC volumes are consistent with prior studies. Lower-SES has been associated with worse working memory (Herrmann and Guadagno, 1997; Farah et al., 2006; Noble et al., 2007; Evans and Schamberg, 2009; Sarsour et al., 2011; Hackman et al., 2015), smaller hippocampal volumes (Hanson et al., 2011; Noble et al., 2012, 2015), and smaller DLPFC volumes (Lawson et al., 2013; Mackey et al., 2015; Noble et al., 2015).

The same MRI data were examined in a whole-brain analysis relating cortical thickness to the income-achievement gap (Mackey et al., 2015), but that study did not examine subcortical volumes or specific memory abilities. Mackey et al. (2015) found that the higher-SES adolescents had higher scores on statewide tests of academic achievement and greater cortical thickness in all lobes of the brain than the lower-SES adolescents. The differences in cortical thickness by SES accounted for almost half of the income-achievement gap. The current study extends the prior findings by showing that SES does not have a global influence on brain and behavior, but rather affects some structures and functions more than others.

One potential explanation for the selective effect of SES on working vs. procedural memory is the differential developmental course of these two memory systems. Working memory develops slowly, continuing to mature into young adulthood (Hale et al., 1997; Gathercole, 1999; Klingberg et al., 2002). This slow development may render the neural systems underlying working memory susceptible to environmental influences, such as the chaotic home environment and poor school quality often associated with lower-SES (Evans, 2004). Procedural memory, on the other hand, develops early (Meulemans et al., 1998; Thomas and Nelson, 2001; Amso and Davidow, 2012). Indeed, 10-year-olds have shown adult-like learning on the probabilistic classification task despite lower levels of performance than adults on measures of complex working memory capacity and declarative memory (Finn et al., accepted). This early development may render the neural systems underlying procedural memory less vulnerable to environmental influences. Thus, SES may not have the same negative impact on procedural memory as it does on working memory due to the differential rate of maturation of these two memory systems.

Another possible explanation of the selective effect of SES on working vs. procedural memory is the association between lower-SES and higher exposure to stress (Baum et al., 1999; Lupien et al., 2001; Chen et al., 2006; McEwen and Tucker, 2011; although we did not measure stress). Both human and animal research have shown that stress negatively impacts the hippocampus and DLPFC (Sapolsky, 1996; Kim and Yoon, 1998; Arnsten, 2009). Indeed, chronic stress in rodents actually enhances the use of signal-response striatal learning and increases neuronal growth in dorsolateral striatum, imperative for habit formation (Kim et al., 2001; Dias-Ferreira et al., 2009; Schwabe and Wolf, 2013). Thus, stress may selectively impair hippocampus and PFC but spare striatal structure and function.

This differential effect of stress on particular brain regions may be especially meaningful for probabilistic classification task performance. Neuroimaging indicates that the probabilistic classification task engages both MTL and striatal systems in a competing fashion (Poldrack et al., 1999, 2001; Foerde et al., 2006). Both rodent and human studies have shown that stress biases learning to be caudate-based rather than hippocampal-based (Kim et al., 2001; Schwabe and Wolf, 2013). For probabilistic classification in particular, neuroimaging indicates that in experimentally stressed participants, learning on this task is correlated with caudate activation, while in non-stressed participants, learning is correlated with hippocampal activation. Strikingly, overall learning was equivalent in both groups (Schwabe and Wolf, 2012). Thus, it is possible that effective caudate-based procedural learning was available to all participants in our study regardless of differential exposure to stress in the two groups.

Although there is compelling lesion evidence that striatum-dependent procedural memory is dissociable from declarative memory in general, and for probabilistic classification in particular (Knowlton et al., 1996), there are many ways in which the DLPFC-MTL and striatal memory systems interact. In regards to working memory, the striatum appears to play a role because patients with striatal dysfunction due to Parkinson's disease have reduced complex working memory capacity (Gabrieli et al., 1996). Further, there is evidence that the basal ganglia play a distinctive role in working memory relative to DLPFC (Awh and Vogel, 2008; McNab and Klingberg, 2008; Voytek and Knight, 2010).

In terms of the probabilistic-learning measure of procedural memory, both MTL and striatal systems appear to be engaged in different ways. In one study, amnesic patients showed intact early learning, but reduced later learning (Knowlton et al., 1994), suggesting that over time learning may shift from a procedural-striatal basis to a declarative-MTL basis. Functional neuroimaging has also shown that probabilistic learning engages both striatal and MTL systems, but indicates that striatal activation is tightly linked to feedback-based learning (Poldrack et al., 1999, 2001; Seger and Cincotta, 2005), whereas MTL activation is related to paired-association learning (Poldrack et al., 2001). This distinction is supported by evidence from lesion studies where Parkinson's patients perform well on a non-feedback based version of the probabilistic classification task (Shohamy et al., 2004) and hypoxic amnesic patients, with specific damage to the hippocampus, showed impaired learning on the feedback based version of this task (Hopkins et al., 2004).

Although the lesion literature provides a conceptual framework for how separable neural systems support different kinds of learning, the influence of a higher- or lower-income environment on both brain and behavior is quite different from a neurological disease. The patient literature involves acute or late-onset degenerative lesions that often affect most of a particular brain region. SES in childhood involves the more subtle development of multiple neural circuits. Thus, although lower-SES adolescents have reduced working memory scores and MTL and DLPFC volumes relative to higher-SES adolescents, they have substantial memory abilities that are far higher than those of amnesic patients. Consequently, lower-SES adolescents can utilize significant declarative and working memory abilities to support learning, including probabilistic learning. Furthermore, there may be interactions between striatal regions and MTL and DLPFC across development. Studies examining a broader age range could examine such developmental interactions among SES, brain volumes, and learning abilities.

The current study has some limitations. First, we report null results, showing a lack of differences in probabilistic classification learning or caudate volumes between SES groups (although this problem has applied to all examples of spared learning in patients with brain lesions). For the procedural learning, this concern is mitigated by the fact that the lower-SES group showed slightly better (although not significant) learning than the higher-SES group. For the brain measures, there were significantly reduced DLPFC and hippocampal volumes in the lower-SES group, but the caudate volumes were also somewhat (albeit non-significantly) smaller in the lower-SES group. Further studies should probe this result in a larger sample. Second, the current study lacked brain volume–behavior relationships. This could be due to small sample sizes within each SES group and should be explored in future studies with larger samples. Third, the current study only explored one measure of procedural memory, but there are multiple kinds of procedural memory that depend on a range of neural structures outside of the striatum, such as cerebellum and other neocortical regions (Gabrieli, 1998). Thus, future studies should probe the range and limits of procedural learning broadly that are unaffected by SES. Fourth, although the complex working memory measure has the advantage of being sensitive to both DLPFC and MTL integrity, a future study could employ separate measures of working memory and conventional declarative memory (delayed recall and recognition without additional cognitive demands). Finally, we lacked measures of stress in our participants, which precludes direct evidence that stress was a critical mechanism underlying the dissociation between different forms of memory as opposed to other factors, such as environmental enrichment, that are also correlated with SES.

Knowledge of the scope and limits of memory abilities in lower-SES adolescents could be of interest for two reasons. First, such knowledge could suggest how environmental variables differentially influence the development of specific memory systems. Second, there is concern about the growing income-achievement gap, the difference in academic achievement between students from higher- and lower-income backgrounds (Reardon, 2011). The findings here raise the possibility that SES may not impair procedural learning, and such learning may be an important resource for individuals from lower-SES backgrounds. At the same time, much of education is targeted toward the accumulation of knowledge that is supported by hippocampal-declarative systems, so it is unclear as to how much a potentially unaffected striatal-procedural system could support school learning.

Perhaps valuable interventions would be those that could enhance hippocampal volume and declarative memory. Although no study has shown hippocampal volume growth in an educational intervention with children, studies of exercise have provided evidence for plasticity in hippocampal volume and declarative memory processes most associated with the hippocampus (relational memory; Cohen and Eichenbaum, 1993; Davachi, 2006). In older adults, an aerobic exercise intervention increased hippocampal volume (Erickson et al., 2011). In children, greater aerobic fitness has been associated with better relational memory (Chaddock et al., 2011) and larger hippocampal volume (Chaddock et al., 2010), and an aerobic fitness intervention enhanced relational memory (Monti et al., 2012). Thus, hippocampal plasticity in children may arise from effective educational interventions. Future research may reveal whether the enhancement of education in lower-SES children is best achieved by exploiting the strengths of their striatal-dependent procedural memory, ameliorating the weaknesses of their hippocampal-dependent working memory, or both.

### Conflict of interest statement

The authors declare that the research was conducted in the absence of any commercial or financial relationships that could be construed as a potential conflict of interest.
